# Enteric vaccines for the developing world: challenges and prospects

**Published:** 2011-01-10

**Authors:** Cecil Czerkinsky

**Affiliations:** 1 International Vaccine Institute, Seoul, Korea

## 

Diarrhea is one of the top disease killers in newborns and young infants of the developing world, yet the research funding for vaccine development of enteric diseases falls far behind the investment that has been made in preventing diseases with lower burden to the world’s population. Advances in this field have been delayed not only due to poor funding, but also due to knowledge gaps in our understanding of the gut mucosal immune system, especially its development during the early life.

Modern biotechnology has yielded an abundance of vaccine candidates against enteric infections. If these candidates are to ultimately reach those in need in developing countries, several lessons from clinical and field research done in these settings must be considered. These lessons include the need to develop vaccines that can be administered without needles or sophisticated delivery devices. These vaccines must work in the most impoverished populations and must be able to contain epidemics following complex emergencies. An ideal vaccine should prevent entry and replication of pathogens in the gut epithelium and confer herd protection. Such vaccine must protect all age groups at high risk and be safe and effective in immunocompromised people.

Extensive research is being carried out to identify protective antigens, pertinent vaccination regimens including prime-boost strategies and alternate delivery routes, novel delivery systems, new vectors and safe adjuvants for mucosal immunization. We have now documented the effectiveness of an oral killed whole cholera bacteria vaccine in children from a cholera endemic area in one of the largest clinical efficacy trials ever conducted for an enteric vaccine. The vaccine can be produced at industrial scale, is affordable to the poorest, and confers direct and community protection for at least 3 years. A second vaccine against typhoid fever is at a late stage of development and has been shown to be highly immunogenic in children. A third vaccine is at an early stage of development and targets *Shigella* bacteria, the causative agent of one of the most severe forms of diarrheal disease, bacillary dysentery. The vaccine is based on a protein antigen conserved among all species and serotypes (>50) of *Shigella* co-administered with a novel mucosal adjuvant and has already been shown to confer protection against experimental shigellosis in 3 animal models. In partnership with PATH, the IVI is conducting a program entailing production process development and early clinical testing of this subunit vaccine anticipated to commence in 2012.

